# Gearbox Fault Diagnosis Based on Improved Variational Mode Extraction

**DOI:** 10.3390/s22051779

**Published:** 2022-02-24

**Authors:** Yuanjing Guo, Shaofei Jiang, Youdong Yang, Xiaohang Jin, Yanding Wei

**Affiliations:** 1 Zhijiang College, Zhejiang University of Technology, Shaoxing 312030, China; yydong@zjut.edu.cn; 2 College of Mechanical Engineering, Zhejiang University of Technology, Hangzhou 310023, China; jsf75@zjut.edu.cn (S.J.); xhjin@zjut.edu.cn (X.J.); 3 Key Laboratory of Advanced Manufacturing Technology of Zhejiang Province, College of Mechanical Engineering, Zhejiang University, Hangzhou 310027, China; weiyd@zju.edu.cn

**Keywords:** gearbox, variational mode extraction, short-time Fourier transform, SDE index, fault feature extraction

## Abstract

Gearboxes are widely used in drive systems of rotating machinery. The health status of gearboxes considerably influences the normal and reliable operation of rotating machinery. When a gearbox experiences tooth failure, a vibration signal with impulse features is excited. However, these impulse features tend to be relatively weak and difficult to extract. To solve this problem, a novel approach for gearbox fault feature extraction and fault diagnosis based on improved variational mode extraction (VME) is proposed. Since the initial value of the desired mode center frequency and the value of the penalty parameter in VME must be assigned, a short-time Fourier transform (STFT) was performed, and a new index, the standard deviation of differential values of envelope maxima positions (SDE), is proposed. The feasibility and effectiveness of the proposed approach was verified by a simulation signal and two datasets associated with a gearbox test bench. The results demonstrate that the VME-based approach outperforms the variational mode decomposition (VMD) approach.

## 1. Introduction

Gearboxes are widely used in rotating machinery, such as wind turbines, nuclear power units, high-speed rail, and new energy vehicles, and their performance influences the normal and reliable operation of rotating machinery. Gearboxes usually operate under a dynamic load or in overload conditions and are prone to suffer from various kinds of defects, such as fatigue pitting, wear, tooth spalling, and tooth fracture [1,2,3]. To evaluate the operating status of rotating machinery and ensure safe production and effective maintenance, it is important to monitor the gearbox condition and diagnose faults. When a gearbox experiences tooth failure, the contact stiffness at the damage point changes, and the meshing movement is not smooth during operation, exciting impulsive vibration. When rotating machinery operates at a constant speed, impulsive vibration can be demonstrated by periodic impulse features in the sampled vibration signal [4,5]. The impulse feature frequency is closely related to the health status of the gearbox, which suggests that different frequencies indicate different fault states [6]. Therefore, extracting the fault feature frequency from the sampled vibration signal is a feasible solution for gearbox fault diagnosis [7]. However, gearboxes usually consist of multiple rotating parts. The vibrations excited by different parts are coupled to each other. In particular, the transient impulses caused by the damaged part attenuate along the transmission path from the source to the sensor, which is usually located on the casing. In addition, the sampled vibration signal often contains environmental noise [8]. Therefore, extracting the weak impulse features from vibration signals with heavy noise becomes a challenging task in practice [9,10,11].

Essentially, the process of fault feature extraction comprises the elimination of noise and interference components in vibration signals. An effective approach to solve this problem is signal decomposition, the variants of which include wavelet decomposition (WT) [12,13,14], empirical mode decomposition (EMD) [11,15,16], local mean decomposition (LMD) [9,10,17,18], and empirical wavelet transform [19,20,21,22]. However, WT is not a self-adaptive signal analysis method because it is restricted by the selection of the wavelet basis function and number of decomposition levels [17]. Although EMD can self-adaptively decompose a multimodulated signal into a series of intrinsic mode functions (IMFs), it lacks a theoretical basis and has some inherent defects, such as sensitivity to noise, end effects, and mode mixing [4,5,15,16]. Like EMD, LMD adaptively decomposes a multicomponent signal into several single-component AM–FM signals but encounters several technical problems, such as end effects and mode mixing [17,18]. EWT decomposes a signal into several AM–FM monocomponents that have compact support in the Fourier spectrum; however, the boundaries of the frequency partition are difficult to set, and this aspect may result in inaccurate and even invalid components [21,22].

In 2014, Dragomiretskiy and Zosso proposed a novel signal processing method known as variational mode decomposition (VMD) [23]. Based on complete mathematical principles, VMD decomposes a signal into an ensemble of band-limited subsignals, i.e., modes. The mode compact around a center frequency is estimated online in a nonrecursive manner. VMD is highly effective when applied to machinery fault diagnosis [5,24,25]. However, the actual performance of VMD is critically affected by the mode number and quadratic penalty parameter. Presetting the two parameters usually requires experience and experimentation. Moreover, it is difficult to determine optimal preset values. To address these limitations, many optimization algorithms, such as genetic optimization [26], particle swarm optimization [25,27], grasshopper optimization [24,28], gray wolf optimization [29,30], cuckoo search algorithm [31], artificial bee colony algorithm [32], and chaotic pigeon-inspired optimization [33], have been studied and applied to achieve the optimal values for the two parameters. Many parameter-optimized or -enhanced VMD methods have been proposed. However, these optimization methods require a large number of iterative operations and are time-consuming, resulting in a low computational efficiency [1], especially given the quadratic penalty parameter usually takes a large value, and its optimal value needs to be searched in a large range. Therefore, the enhanced VMD method combined with several optimization algorithms must be further studied and discussed to enhance its practicality.

In gearbox fault-diagnosis applications, the fault feature information is usually included in a certain frequency band of the sampled vibration signal. Therefore, among the obtained modes of VMD, only one invaluable target mode exists, and the other modes may not be as important. Selection of the target mode is challenging because some experience and knowledge of fault diagnosis is required, or several indicators, such as kurtosis [26], weighted kurtosis index [24,29,32], Shannon entropy [34], and sample entropy [35], must be considered.

In 2017, Nazari and Sakhaei proposed a new efficient method known as variational mode extraction (VME) to derive respiratory signals from ECG [36]. VME, which is based on a similar principle as that of VMD, directly decomposes a signal into two modes, i.e., the desired mode and residual signal. Therefore, VME resolves the difficulty of determining the mode number and selecting the target mode associated with VMD. Nevertheless, the application of VME necessitates the consideration of two key aspects. First, the initial center frequency of the desired mode must be correctly preset according to the analyzed signal. Otherwise, the desired mode may not contain valuable fault-related information. Second, the penalty parameter must be set to an appropriate value. If the penalty parameter value is excessively small, considerable noise and interference may remain in the desired mode. Moreover, the penalty parameter value must not be excessively large; otherwise, the desired mode may lose useful information.

Considering these aspects, this paper proposes an improved VME method, in which the initial value of the desired mode center frequency is selected by the STFT spectrum, and the optimal value of the penalty parameter is determined using a novel index: the standard deviation of differential values of envelope maxima positions (SDE). The subsequent sections of this paper are organized as follows. The VME method is briefly introduced in Section 2. Section 3 describes the effect of the initial value of the desired mode center frequency and the penalty parameter value on the performance of VME, as well as the methods to determine these parameters. The improved VME method for gearbox fault diagnosis is summarized in Section 4. Two cases of gearbox fault vibration signal analysis based on the improved VME method are described in Section 5. Concluding remarks are presented in Section 6.

## 2. Variational Mode Extraction

### 2.1. Basic Theory

The basic principle of VME is similar to that of VMD; however, VME explicitly decomposes an original signal, *x*(*t*), into two modes: the desired mode, *u*_d_(*t*), and residual signal, *x*_r_(*t*), as indicated in Equation (1):

*x*(*t*) = *u*_d_(*t*) + *x*_r_(*t*) (1)

where *u*_d_(*t*) is a compact function around the center frequency, *ω*_d_. In the application of rotating machinery fault diagnosis, *u*_d_(*t*) denotes the fault feature component. To extract *u*_d_(*t*) from *x*(*t*), the spectral overlap between the two components must be minimized. In other words, *x*_r_(*t*) has the minimal energy in the frequency band of *u*_d_(*t*), particularly zero energy in the center frequency, *ω*_d_ of *u*_d_(*t*). Moreover, the sum of *u*_d_(*t*) and *x*_r_(*t*) must completely reconstruct the original signal, *x*(*t*). Therefore, the mathematical model of the VME method can be expressed by the constrained minimization problem in Equation (2):

(2) $$\begin{array}{cc} \underset{u_{d},\omega_{d},f_{r}}{\min} & \left\{\alpha {\left\|\partial_{t}\left[\left(\delta (t)+\frac{j}{\pi t}\right)*u_{d}(t)\right]e^{-j\omega_{d}t}\right\|}_{2}^{2}+{\left\|\beta (t)*x_{r}(t)\right\|}_{2}^{2}\right\}\\ s.t. & u_{d}(t)+x_{r}(t)=x(t) \end{array}$$

where ∂*_t_* denotes the partial derivative with time, *t*; *δ*(*t*) is the Dirac delta distribution; and * is the convolution operator. *β*(*t*) represents the impulse response of the filter, which is used to filter the frequencies in *x*_r_(*t*) overlapping with *u*_d_(*t*) and can be described as in Equation (3):

(3) $$\hat{\beta }(\omega )=\frac{1}{\alpha {\left(\omega -\omega_{d}\right)}^{2}}$$

Combining Equations (2) and (3), it can be observed that the penalty parameter, *α*, plays a critical role in controlling the balance between the compactness of *u*_d_(*t*) and spectral overlap between *u*_d_(*t*) and *x*_r_(*t*).

To solve the constrained minimization problem, the Lagrangian multiplier, *λ*(*t*), is introduced. Hence, the constrained minimization problem defined in Equation (2) can be converted into an unconstrained optimization problem, as expressed in Equation (4):

(4) $$\begin{array}{ll} L\left(u_{d},\omega_{d},\lambda \right) & =\alpha {\left\|\partial_{t}\left[\left(\delta (t)+\frac{j}{\pi t}\right)*u_{d}(t)\right]e^{-j\omega_{d}t}\right\|}_{2}^{2}+{\left\|\beta (t)*x_{r}(t)\right\|}_{2}^{2}\\ & +{\left\|x(t)-\left(u_{d}(t)+x_{r}(t)\right)\right\|}_{2}^{2}+\langle \lambda (t),x(t)-\left(u_{d}(t)+x_{r}(t)\right)\rangle \end{array}$$

According to Parseval’s theorem, the optimization problem presented in Equation (4) can be rewritten in the frequency domain as in Equation (5):

(5) $$\begin{array}{ll} L\left(u_{d},\omega_{d},\lambda \right) & =\alpha {\left\|j(\omega -\omega_{d})\left[\left(1+\mathrm{sgn}(\omega )\right)\cdot {\hat{u}}_{d}(\omega )\right]\right\|}_{2}^{2}+{\left\|\hat{\beta }(\omega )\cdot {\hat{x}}_{r}(\omega )\right\|}_{2}^{2}\\ & +{\left\|\hat{x}(\omega )-\left({\hat{u}}_{d}(\omega )+{\hat{x}}_{r}(\omega )\right)\right\|}_{2}^{2}+\langle \hat{\lambda }(\omega ),\hat{x}(\omega )-\left({\hat{u}}_{d}(\omega )+{\hat{x}}_{r}(\omega )\right)\rangle \end{array}$$

The alternating direction method of multipliers (ADMM) is adopted to solve Equation (5) to identify the saddle point of the augmented Lagrangian expression. As the optimization problem is converted into a series of iterative suboptimization problems solved by ADMM, the updating equations, in terms of ${\hat{u}}_{d}$, $\omega_{d}$, and $\hat{\lambda }$ are expressed as Equations (6)–(8), respectively:

(6) $${\hat{u}}_{d}^{n+1}(\omega )=\frac{\hat{x}(\omega )+\alpha^{2}{\left(\omega -\omega_{d}^{n+1}\right)}^{4}\cdot {\hat{u}}_{d}^{n}(\omega )+\hat{\lambda }(\omega )/2}{\left[1+\alpha^{2}{\left(\omega -\omega_{d}^{n+1}\right)}^{4}\right]\left[1+2\alpha {\left(\omega -\omega_{d}^{n}\right)}^{2}\right]}$$

(7) $$\omega_{d}^{n+1}=\frac{\int_{0}^{\infty }\omega {\left|{\hat{u}}_{d}^{n+1}(\omega )\right|}^{2}d\omega }{\int_{0}^{\infty }{\left|{\hat{u}}_{d}^{n+1}(\omega )\right|}^{2}d\omega }$$

(8) $${\hat{\lambda }}^{n+1}={\hat{\lambda }}^{n}+\tau \cdot \left[\frac{\hat{x}(\omega )-{\hat{u}}_{d}^{n+1}(\omega )}{1+\alpha^{2}{\left(\omega -\omega_{d}^{n+1}\right)}^{4}}\right]$$

where ${\hat{u}}_{d}^{n}(\omega )$ is the obtained desired mode in the *n*th iteration with the center frequency $\omega_{d}^{n}$, $\hat{x}(\omega )$ represents the original signal Fourier transforms, *n* is the number of iterations, and τ is the iteration step length. The steps involved in VME are as follows [36].

Step 1: initialize ${\hat{u}}_{d}^{1}$, $\omega_{d}^{1}$, ${\hat{\lambda }}^{1}$, and *n* = 1.

Step 2: update ${\hat{u}}_{d}$ and $\omega_{d}$ according to Equations (6) and (7), respectively.

Step 3: update $\hat{\lambda }$ according to Equation (8).

Step 4: *n* = *n* + 1; repeat Steps 2–3 until the iteration termination condition,

(9) $$\frac{{\left\|{\hat{u}}_{d}^{n+1}-{\hat{u}}_{d}^{n}\right\|}_{2}^{2}}{{\left\|{\hat{u}}_{d}^{n}\right\|}_{2}^{2}}<\varepsilon ,$$

is satisfied.

Step 5: obtain the desire mode, *u_d_*(*t*).

### 2.2. Analysis of Parameter Influence

In the implementation of VME, the update step size, *τ*, is often set as zero to ensure that the algorithm converges effectively [36]. The value of the convergence tolerance, *ε*, determines the reconstruction accuracy of the VME decomposition; therefore, this parameter is usually set as an extremely small positive value (e.g., 1 × 10^−6^ in our experiments). The performance of VME is significantly dependent on the decomposition parameters, such as the initial center frequency, *ω*_d_, of the desired mode and penalty parameter *α*, which should be preset in a restrained manner. The effects of these parameters on VME performance are analyzed below.

(1) Initial center frequency, *ω*_d_, of the desired mode. The frequency band position of the desired mode extracted from the original signal is determined by the initial value of *ω*_d_. If the initial value of *ω*_d_ is inappropriately initialized, the desired mode likely does not contain valuable information. Although VME is not highly sensitive to the initial *ω*_d_ value, which can be preset in a wide range [36], the approximate range of the initial *ω*_d_ value must be reasonably determined.
(2) Penalty parameter *α*. As this parameter controls the compactness of the desired mode, it also determines the degree of spectral overlap between the desired mode and residual signal. When *α* is extremely small, the bandwidth of the desired mode is extremely large; therefore, lots of interference components or additional noise may be included in the desired mode, which can hinder the identification of valuable information. Normally, parameter *α* is necessarily set to a large value to ensure that the detected center frequency is closely related to the desired mode [36]. However, when *α* is set to an extremely large value, the bandwidth of the desired mode is extremely small, and part of the useful information may be lost, especially when the center frequency, *ω*_d_, is not appropriately set. Consequently, the penalty parameter, α, must be preset to an appropriate value.

## 3. Simulation Analysis and Discussion

### 3.1. Simulation Signal Construction

This section describes the construction of a simulation vibration signal to evaluate the effects of the initial center frequency, *ω*_d_, and penalty parameter, *α*, on the fault feature extraction performance of VME. Considering these effects, methods to determine the two parameters are established.

Consider a pair of engaged gears, in which one of the gears is assumed to have a local damage fault. When the gears are operating smoothly, the fault vibration signal, *s*(*t*), can be considered to be composed of three main components, including the multiple AM–FM components, *s*_m_(*t*), induced by gear meshing and shaft rotation; a periodic impulsive component, *s*_p_(*t*), excited by gear local damage; and additive noise, *s*_n_(*t*). The mathematical model of the fault vibration signal, *s*(*t*), can be presented as:

(10) $$s(t)=s_{m}(t)+s_{p}(t)+s_{n}(t)$$

(11) $$s_{m}(t)=\sum_{n=1}^{N}X(n)\left[1+a_{n}(t)\right]\cos\left[2\pi nZf_{r}t+\varphi_{n}+b_{n}(t)\right]$$

(12) $$s_{p}(t)=\sum_{k=1}^{K}P(k)e^{-\zeta (t-kT)}\cos\left[2\pi Zf_{r}(t-kT)\right]$$

where *Z* and *f*_r_ are the number of teeth and rotation frequency of the damaged gear, respectively; *Z*·*f*_r_ denotes the meshing frequency of the gears, represented by *f*_m_, i.e., *f*_m_ = *Z*·*f*_r_; *N* is the number of AM–FM components; *X*(*n*), *φ_n_*, *a*_n_(*t*), and *b*_n_(*t*) denote the amplitude, initial phase, amplitude-modulation function, and phase-modulation function of the *n*th AM-FM component, respectively; and *K*, *ζ*, and *T* denote the number, amplitude-damping coefficient, and period of impulses, respectively. When the damage gear rotates once, the fault impulse feature is triggered once; thus, *T* = 1/*f*_r_, and the fault feature frequency, *f*_3f_ = *f*_r_. *P*(*k*) is the amplitude of the *k*th impulse. Furthermore, the modulation functions, *a*_n_(*t*) and *b*_n_(*t*), can be described as:

(13) $$\left\{\begin{array}{c} a_{n}(t)=A_{n}\cos(2\pi nf_{r}t+\alpha_{n})\\ b_{n}(t)=B_{n}\cos(2\pi nf_{r}t+\beta_{n}) \end{array}\right.$$

where *A_n_* and *α_n_* denote the amplitude and phase of the amplitude-modulation function, respectively, and *B_n_* and *β_n_* denote the amplitude and phase of the phase-modulation function, respectively. The fault vibration signal of this pair of gears can be simulated by setting the appropriate values for the relevant parameters. The values of all parameters in the simulated signal are listed in Table 1.

**Figure 1:**
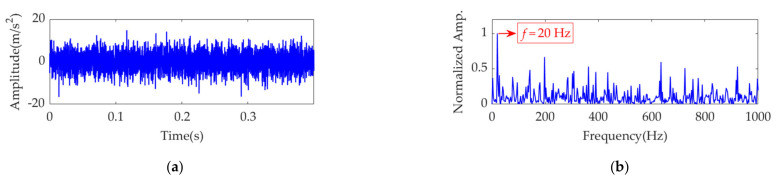
The simulation signal: (**a**) time–domain waveform and (**b**) SES.

The sampling frequency, *f*_s_, is set as 10 k Hz, and the sampling time, *t*_s_, is set as 0.4 s. Therefore, the number of sampling points is 4000. In the sampling period, the first impulse feature is assumed to appear at *t*_1_ = 0.025 s, and the number of impulses is *K* = ⌊(*t*_s_ − *t*_1_)·*f*_r_⌋, where ⌊·⌋ denotes the floor function. The amplitude sequences, *P*(*k*) (*k*=1,2, …, 8), for the impulses are obtained from a Gaussian distribution with a mean of 1.3 and standard deviation of 0.2. White Gaussian noise is introduced such that the signal-to-noise ratio (SNR) of the simulated signal is −8 dB. The time-domain waveform of the simulation signal, *s*(*t*), is shown in Figure 1a. As the impulsive component is almost buried in the strong background noise, it is difficult to detect the fault by observing the amplitude of the simulation signal. The square envelope spectrum (SES) [37] of the simulation signal, *s*(*t*), is shown in Figure 1b, in which the *Y*-axis is the normalized amplitude obtained by dividing each amplitude by the maximum amplitude. From the SES, the fault feature frequency can be extracted, although it is easily disturbed by other frequency contents with large amplitudes. VME is applied to extract the weak fault features from the simulation signal, and further analysis is performed.

### 3.2. Initial Value Estimation of ω_d_

As mentioned, the fault feature extraction effect of VME is highly dependent on the initial value of the desired mode center frequency, *ω*_d_. An appropriate initial value of *ω*_d_ can help ensure that the desired mode contains complete and relatively pure fault features. In this study, the initial value of *ω*_d_ is selected using a short-time Fourier transform (STFT).

STFT is a classical method for joint time–frequency analysis that is suitable for not only stationary signals but also time-varying, nonstationary signals. The basic idea of STFT is to multiply the original signal by a fixed-length window function, assuming that the signal section in the short time interval of the analysis window is stationary, and to implement a Fourier transform (FT) for the signal section to obtain a local spectrum. By translating the analysis window along the entire time horizon, the original signal is analyzed by FT in a section-by-section manner, and a set of local spectra is obtained during each time interval. Therefore, the basic calculation formula of STFT can be expressed as:

(14) $$STFT\left\{x(t)\right\}(t,f)=\int_{-\infty }^{+\infty }x(\tau )g(\tau -t)e^{-j2\pi f\tau }d\tau$$

where *x*(*t*) is the original signal, *t* and *τ* denote the time, *f* denotes the frequency, and *g*(*τ* − *t*) is a window function with the center at time *t*. For a given time, *t*, *STFT*(*t*, *f*) can be considered to be the FT spectrum. In particular, when the window function is set as 1, the STFT degenerates into the traditional FT.

**Figure 2:**
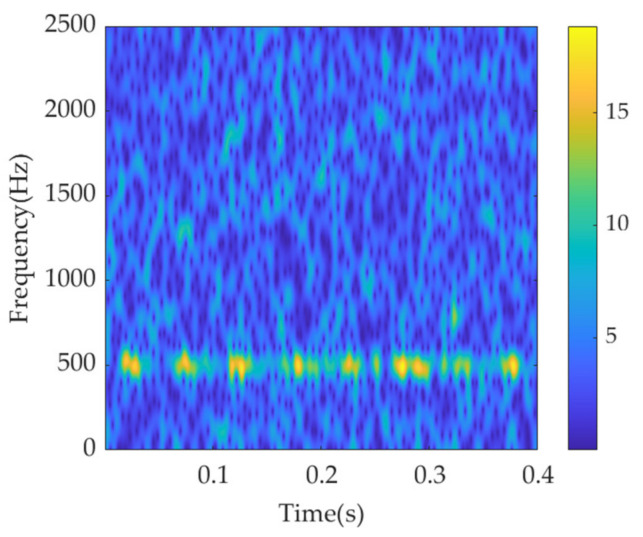
STFT spectrum of the simulation signal.

To obtain relatively optimal localization performance, the type and width of the window function must be selected appropriately according to the signal characteristics. In this study, considering the exponential attenuation tendency of the transient impulse feature, the Gaussian window function is applied, and its length is set as 65 for a high time resolution. The time–frequency spectrum of the simulated signal by STFT is shown in Figure 2. Because of the heavy noise, the morphology and periodicity of the impulses undergo considerable interference. Nevertheless, it can be seen that the impulses repeatedly appear in a narrow frequency range centered at 500 Hz along the time axis. Therefore, the initial center frequency, *ω*_d_, is set as 2π·500 rad/s, which can also be considered an optimal value.

Because a reliable basis for the selection of the penalty parameter, *α*, is not available, the *α* value is set as 10,000, 80,000 and 150,000. Experimental results indicate that it is reasonable to set *α* as 80,000 because the desired mode obtained using this value has the fewest interference components, and the fault feature frequency can be successfully extracted. When *α* is extremely small, the spectral overlap between the desired mode and residual signal is large; therefore, the desired mode may contain many noise components. When *α* is extremely large, the desired mode exhibits a high compactness. Thus, useful components may be lost, and the signal may degenerate into a harmonic signal with a frequency of *ω*_d_, from which the fault feature frequency cannot be extracted. Thus, an optimal value must be set for the penalty parameter, *α*, which requires further study.

### 3.3. SDE Index

To investigate the effect of parameter settings on VME performance, it is critical to construct an accurate objective function. The desired mode, *u*_d_(*t*), theoretically obtained by VME contains a series of fault impulse features with as complete a periodicity as possible and as little interference or noise as possible. To assess the periodicity and purity of the desired mode, this paper proposes a new index called the standard deviation of differential values of envelope maxima positions (SDE), which can be determined using the following steps.

Step 1: Demodulate the desired mode, *u*_d_(*t*), using the Hilbert transform to obtain the upper envelope, *ue*_d_(*t*).

Step 2: Find the positions of the local maximum points in *ue*_d_(*t*) that are greater than a certain threshold, *m*_th_, expressed as:

(15) $$m_{\mathrm{th}}=\eta \cdot \left\{\max\left[ue_{d}(t)\right]-\min\left[ue_{d}(t)\right]\right\}$$

where *η* is the threshold coefficient and, in this paper, all the *η* values are set as 0.25. These positions of the target points are denoted by *p_i_*, *i* = 1, 2, …, *N*, where *N* is the number of local maximum points.

Step 3: Calculate the differential values, *d_j_*, of series *p_n_*,

(16) $$d_{j}=p_{j+1}-p_{j},j=1,2,\cdots ,N-1.$$

Step 4: Calculate the standard deviation of series *d_j_*, i.e., SDE, and use it as the indicator of the periodic fault impulse features.

(17) $$SDE=\sqrt{\frac{1}{N-1}\sum_{j=1}^{N-1}{\left(d_{j}-\bar{d}\right)}^{2}}$$

where $\bar{d}$ is the mean value of *d_j_*.

Ideally, regardless of the amplitude differences between the fault impulses, the positions of the maximum amplitudes are uniform due to periodicity, and the calculated SDE index is zero. In practice, the fault impulse features in vibration signals may not have strict periodicity, causing the position intervals of the fault impulses to fluctuate slightly and randomly. Nevertheless, the calculated SDE index is expected to be extremely small due to the randomness of fault impulse occurrence positions. According to the calculation process described above, in an ideal situation, the value of the SDE index is mainly determined by the maximum value of each fault impulse feature. Therefore, the SDE index is adaptive to the periodicity of fault impulse features and can clarify the purity of the fault-related information in the desired mode obtained by VME.

### 3.4. Effect of α

To study the effect of the penalty parameter, *α*, on VME performance and to identify a way to select an optimal value for *α*, the initial center frequency, *ω*_d_, is set as a fixed value of 2π·500 rad/s, and the value of *α* increases gradually from *α*_min_ to *α*_max_ at a step size of *s*_α_. At the nth step, the value, *α_n_*, of the penalty parameter is calculated as:

(18) $$\alpha_{n}=\alpha_{\min}+(n-1)\cdot s_{\alpha },n=1,2,\cdots ,\left(\alpha_{\max}-\alpha_{\min}\right)/s_{\alpha }$$

**Figure 3:**
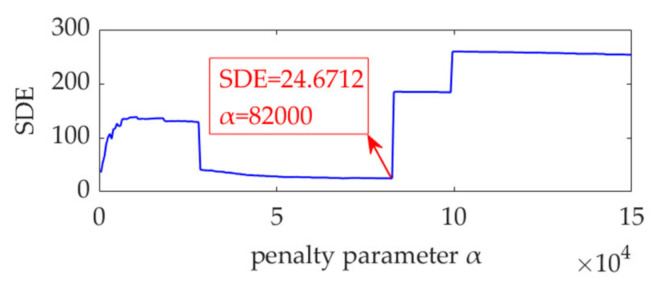
Relationship between the SDE index and penalty parameter *α*.

For the simulation signal, *α*_min_, *α*_min_, and *s*_α_ are set as 500, 150,000, and 500, respectively. VME is implemented with a fixed *ω*_d_ value of 2π·500 rad/s and different *α* values of *α_n_*, *n*=1, 2, ⋯, 299. The SDE index value for each desired mode is calculated. The curve of the SDE index with different penalty parameter, *α*, values is shown in Figure 3.

**Figure 4:**
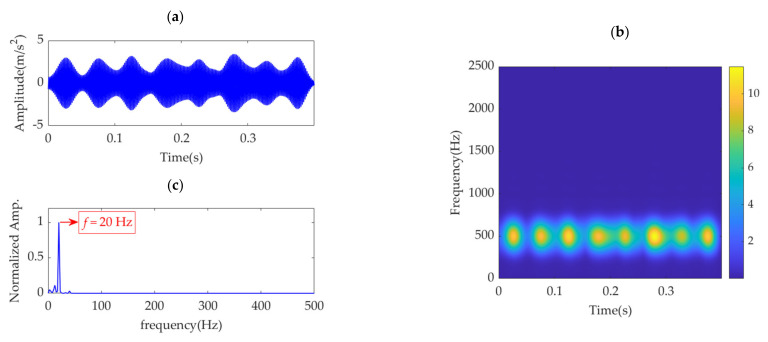
Desired mode obtained by VME with *α*_opt_ = 82,000: (**a**) time−domain waveform, (**b**) STFT spectrum, and (**c**) SES.

In the initial stage of the curve, the SDE index acquires a small value. The main reason for this is that as the value of α is extremely small, the obtained desired mode still contains considerable noise, and the position distribution of the desired mode peaks has strong randomness and uniformity. As the value of α continues to increase, the noise in the obtained desired mode is gradually suppressed, the randomness and uniformity of its peak distribution are broken, and the resulted values of the SDE index also increase. When the noise in the desired mode is eliminated, the SDE index reaches its minimal value due to the periodicity of fault impulse features. However, if the value of α is further enhanced, the fault impulse features in the obtained desired mode will be suppressed, especially those with small amplitudes, and the periodicity of the fault impulse features will be disturbed, leading to nonuniformity of the peaks in the desired mode and a large value of the SDE index. Such an analysis shows that when the SDE index reaches the minimum value of 24.6712, the optimal value of *α* is obtained, expressed as *α*_opt_ = 82,000. VME achieves the optimal effect for fault impulse feature extraction. In other words, the obtained desired mode exhibits minimal interference with the noise and exhibits the most significant periodicity. The desired mode, along with its STFT spectrum, and the extracted fault feature frequency using SES are shown in Figure 4.

In addition, Figure 3 shows that the values of the SDE index vary slightly around *α*_opt_. Within this varying interval, some other values of the SDE index are selected, e.g., *SDE* = 27.3522 and *SDE* = 24.8577, corresponding to *α* = 52,000 and *α* = 68,500, respectively. The numerical experimental results show that the effects of *α* = 52,000 and *α* = 68,500 on the VME performance are basically identical to those of *α*_opt_ = 82,000. Therefore, although there exists an optimal value for *α*, the performance of VME is not highly sensitive to *α* as long as the *α* value is selected within a reasonable range, which can be easily estimated using the *α*–SDE index curve.

### 3.5. Effect of Initial ω_d_

The influence of the penalty parameter, *α*, on VME performance is examined with the initial center frequency *ω*_d_ set as 2π·500 rad/s, as obtained using the STFT. Moreover, the optimal value of *α* is *α*_opt_ = 82,000. Thus, α is set as a fixed value of 82,000, and *ω*_d_ increases from *ω*_dmin_ to *ω*_dmax_, with a step size of *s*_ω_. At the *n*th step, the value of *ω*_d*n*_ is calculated as:

(19) $$\omega_{d}{}_{n}=\omega_{d}{}_{\min}+(n-1)\cdot s_{\omega },n=1,2,\cdots ,\left(\omega_{d}{}_{\max}-\omega_{d}{}_{\min}\right)/s_{\alpha }$$

**Figure 5:**
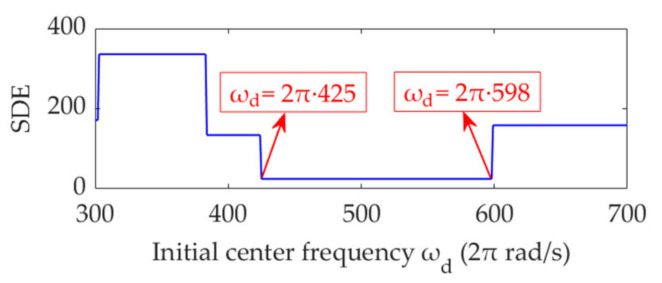
Relationship between the initial center frequency, *ω*_d_, and SDE index.

As shown in Figure 2, periodic fault impulse features appear in a frequency band with the center frequency, *f*_d_ = 500 Hz. To include this frequency band, *ω*_dmin_, *ω*_dmax_, and *s_ω_* are set as 2π·300 rad/s, 2π·700 rad/s, and 2π rad/s, respectively. VME is implemented with initial center frequency, *ω*_d*n*_ (*n* = 1, 2, …, 401), to achieve the desired mode, and the corresponding SDE index value is calculated. The curve of the SDE index with *ω*_d*n*_ is shown in Figure 5.

The SDE index is minimized in the *ω*_d_ range of 2π·425 to 2π·598 rad/s. In this range of *ω*_d_, VME exhibits the same performance for periodic fault impulse feature extraction. In other words, as long as the *ω*_d_ value is estimated within an appropriate range, the performance of VME is not sensitive to *ω*_d_. In practice, the targeted value range of *ω*_d_ can be clarified using the STFT spectrum, and a reasonable or optimal *ω*_d_ value can be easily obtained.

### 3.6. Comparative Study between the SDE Index and Other Indices

**Figure 6:**
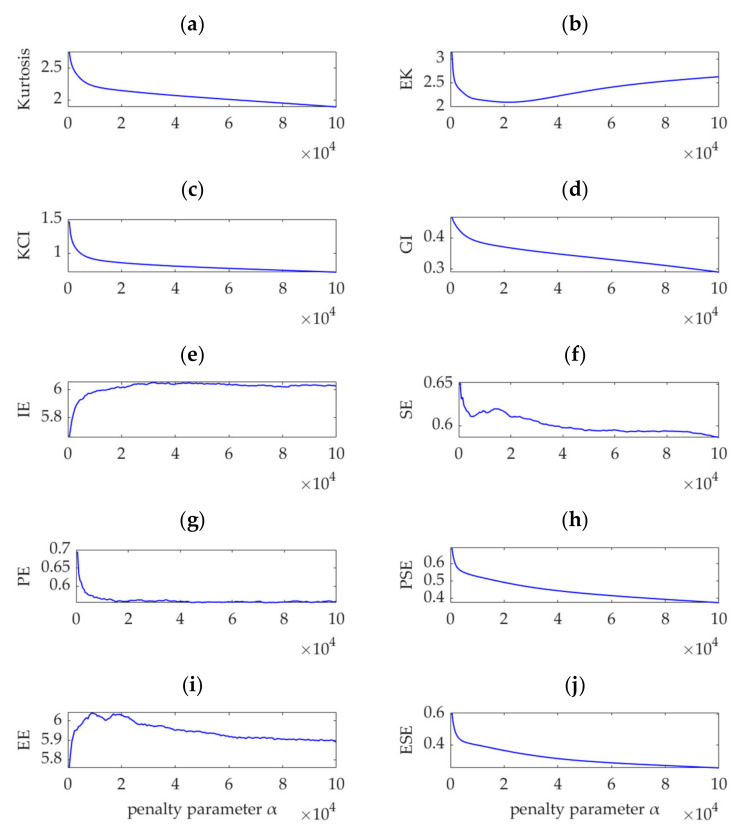
Relationship between different indices and penalty parameter α.

Many indicators have been proposed for impulse detection and fault feature extraction from rotating machinery vibration signals. Examples of widely used indicators are kurtosis [1]; the weighted kurtosis index (also known as KCI) [24,29,32,38]; envelope kurtosis (EK) [39]; the Gini index (GI) [40]; and entropy-class indices, such as information entropy (IE, also known as Shannon entropy) [26,34], sample entropy (SE) [35,41], permutation entropy (PE) [15], power spectral entropy (PSE) [42], envelope entropy (EE) [30], and envelope spectrum entropy (ESE) [43]. To verify the superiority of the proposed SDE index, the values of these 10 indices are calculated for the desired modes obtained by VME with different penalty parameters, *α*_n_ (*n* = 1, 2, …, 300). The relationship between these 10 indices and penalty parameter α are shown in Figure 6.

**Figure 7:**
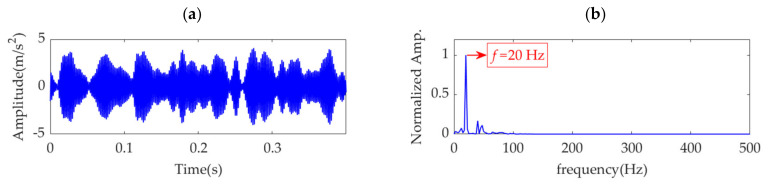
Desired mode obtained by VME with α = 9000: (**a**) time–domain waveform and (**b**) SES.

**Figure 8:**
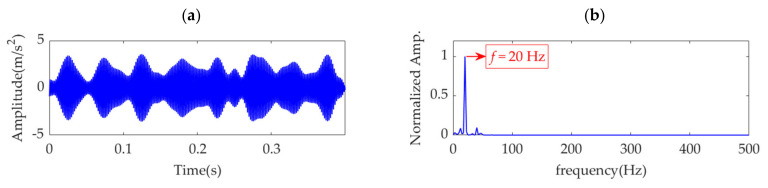
Desired mode obtained by VME with α = 32,500: (**a**) time–domain waveform and (**b**) SES.

**Figure 9:**
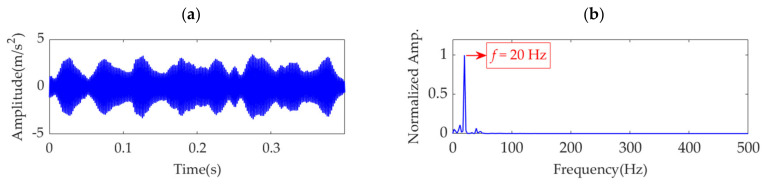
Target mode obtained by VMD: (**a**) time–domain waveform and (**b**) SES.

VME is equivalent to the implementation of a bandpass filter around the center frequency, *ω*_d_, and the penalty parameter, α, determines the passband width of the filter. Theoretically, when *α* is small, considerable noise and other interference components remain in the desired mode. As *α* increases and the compactness of the desired mode intensifies, the noise and interference components are gradually depressed. When *α* increases to a certain value, the optimal fault impulse features are obtained. As *α* further increases, the fault-related features are damped accordingly, and the desired mode tends to be a single-component signal in which the fault-related information may be completely lost. Therefore, an effective index must achieve a significant extremum or sudden change in the search range of *α* to ensure that it can indicate the possible optimal value of *α*. Considering this aspect, the indices of kurtosis, KCI, GI, PE, PSE, and ESE cannot indicate the possible optimal *α* value due to their monotonic changes, as shown in Figure 6a,c,d,g,h,j. Nevertheless, in the search range of *α*, the EK index is minimized, as shown in Figure 6b, and the corresponding value of *α* is 21,500. In contrast, the IE and EE indices are maximized, as shown in Figure 6e,i, and the corresponding values of *α* are 32,500 and 9000, respectively. In addition, Figure 6f shows that the SE index obtains a local maximum value, which indicates *α* to be 14,000. Therefore, the optimal values of *α* are respectively assumed to be 9000 and 32,500, and the obtained desired modes, together with their corresponding square envelope spectra, are shown in Figure 7 and Figure 8, respectively. Comparing Figure 8 and Figure 9 with Figure 5, although the differences between the performances of VME with different optimal α values are not significant, the desired mode obtained through VME with the proposed SDE index has fewer interference components. This outcome verifies the robustness of VME in response to penalty parameter *α* and the superiority of the SDE index compared to other indices in this application.

### 3.7. Comparative Study between VME and VMD

To further evaluate the superiority of VME, the simulation vibration signal is decomposed using VMD. To ensure a fair comparison, the value of penalty parameter *α*_d_ in VMD is the same as the previously obtained optimal value of penalty parameter *α* in VME, i.e., *α*_d_ = 82,000. After several trials, the number of modes in VMD is determinately set as 8. In the VMD results, the target mode that contains fault impulse features is the second mode, as shown in Figure 9a. The SES of the target mode is shown in Figure 9b. Comparison of Figure 4c and Figure 9b shows that the desired mode obtained by VME is similar to the target mode obtained by VMD, although the fault feature frequency can be successfully extracted from either of the two modes. However, compared with VME, the VMD method exhibits several deficiencies:

(1) The exact number of modes is not easy to determine, and the selection of the target mode with valuable fault-related information is challenging to perform simultaneously. These two issues can be solved through trials or an optimization method; however, the execution efficiency of VMD may decrease.
(2) Considerable noise is present in the target mode of VMD, and less noise is present in the desired mode of VME. This phenomenon likely occurs because there exist certain overlaps between the target mode and its previous and latter modes. Therefore, a small amount of noise associated with the previous and latter modes remains in the target mode.

As VME decomposes a signal into two layers, i.e., the desired mode and residual signal, the problems of decomposed mode number determination and target mode selection associated with VMD can be avoided. In addition, the penalty parameter, α, in VME only adjusts the spectrum overlap degree between the desired mode and residual signal. In contrast, the penalty parameter, α_d_, in VMD must balance the spectrum overlap between all adjacent modes. Therefore, typically, the desired mode obtained by VME is purer than the target mode obtained by VMD.

## 4. Improved VME Method for Gearbox Fault Diagnosis

**Figure 10:**
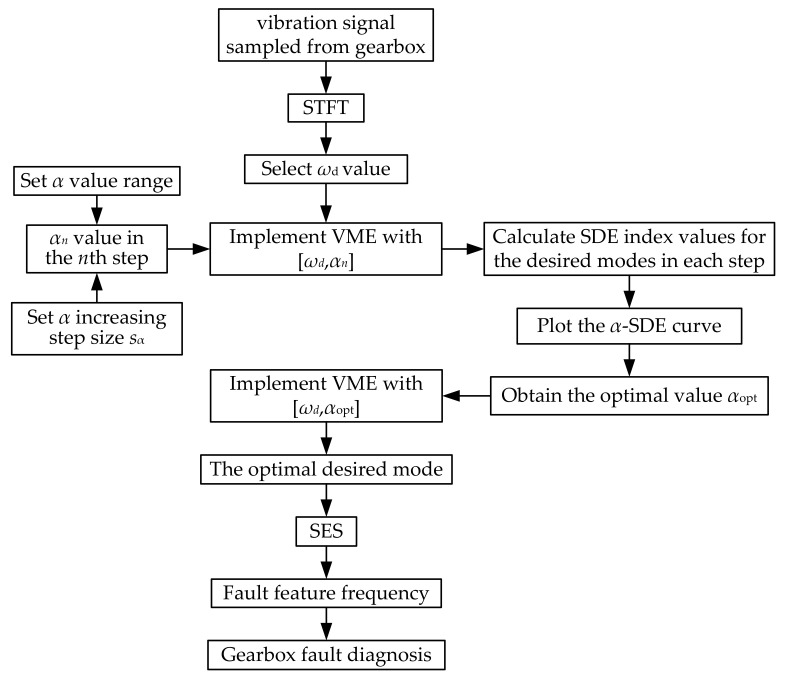
Process flow of gearbox fault diagnosis based on improved VME.

Based on the previous analysis, this paper proposes an improved VME method to extract the fault-related features from vibration signals for gearbox fault diagnosis. The fault diagnosis process is shown in Figure 10. The main implementation steps are described as follows.

Step 1: The time–frequency spectrum of the vibration signal sampled from the gearbox is obtained using STFT to detect the approximate frequency band location of fault-related features. Accordingly, the initial value of the center frequency, *ω*_d_, is selected and considered the optimal value.

Step 2: The value range of penalty parameter *α* is set as [*α*_min_, *α*_max_], and the increasing step size is set as *s*_α_. Subsequently, *α* is increased from *α*_min_. In the *n*th step, the *α* value is expressed as *α*_n_.

Step 3: VME is implemented with parameter group [*ω*_d_, *α_n_*] in each step for the original vibration signal to obtain the desired mode. The corresponding SDE index value is calculated.

Step 4: Once *α* increases to *α*_max_ and the last SDE value is calculated, the α–SDE curve is plotted. Using this curve, the optimal α value is selected as *α*_opt_, corresponding to the minimal SDE index value.

Step 5: VME is implemented with the optimal parameter group [*ω*_d_, *α*_opt_] for the original vibration signal to obtain the optimal desired mode.

Step 6: SES analysis is performed for the optimal desired mode to extract the fault feature frequency and diagnose gearbox faults.

## 5. Experimental Evaluation

As described in this section, two vibration datasets of gearboxes obtained using a wind turbine gearbox simulation test bench are sampled and considered to verify the effectiveness and feasibility of the improved VME method for gearbox fault diagnosis.

### 5.1. Gearbox Test Bench

**Figure 11:**
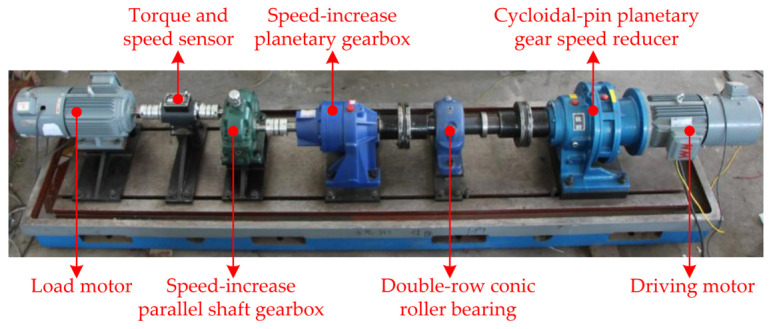
Gearbox test bench.

The gearbox test bench is constructed to simulate the function of the wind turbine gearbox and evaluate the gearbox fault diagnosis methods, shown in Figure 11. The mechanical components in the test bench include the driving motor, cycloidal-pin planetary gear speed reducer, double-row conic roller bearing, two-stage speed-increase planetary gearbox, one-stage speed-increase parallel shaft gearbox, torque and speed sensors (±30 Nm torque measurement range, 1024 line incremental encoder, and ±6000 rpm standard speed measurement range), and load motor. The combination of the driving motor and cycloidal-pin planetary gear speed reducer produces a low speed to simulate the wind turbine main shaft speed. The combination of a two-stage speed-increase planetary gearbox and a one-stage speed-increase parallel shaft gearbox is used to imitate the wind turbine gearbox with the widely used configuration involving two-stage planetary and one-stage parallel shaft gear transmission. In this study, the test object is the parallel shaft gearbox, which simulates the high-speed stage prone to failure in a wind turbine gearbox. The gear ratio, gear modulus, number of gear teeth, and number of pinion teeth of the parallel shaft gearbox are 2.45, 2, 54, and 22, respectively.

**Figure 12:**
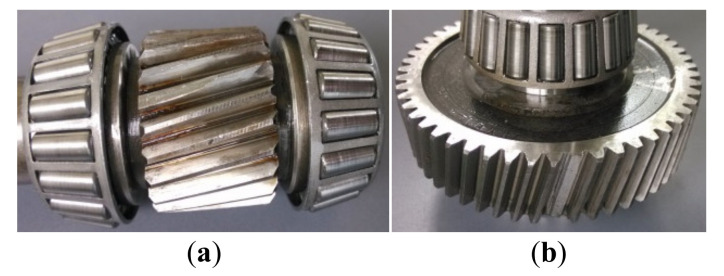
(**a**) Pinion-tooth partial fracture fault and (**b**) gear-tooth fracture fault.

Two types of gear damages, i.e., pinion-tooth partial fracture and gear-tooth fracture, are manually induced in the experimental gearbox, as shown in Figure 12. Subsequently, operational tests are conducted over the test bench for each fault state. In each test, the load motor is set to operate in the power-generation state and output a torque of 3 Nm. An IEPE (integrated-electronics piezoelectric) accelerometer (±50 g full scale range, 100 mV/g sensitivity, and 0.2–5000 Hz frequency range) is mounted on the experimental gearbox top cover using a suitable magnetic mount to acquire the vibration signal. The sampling frequency of vibration signal is set as 5.12 kHz.

### 5.2. Pinion Fault Vibration Dataset Analysis

**Figure 13:**
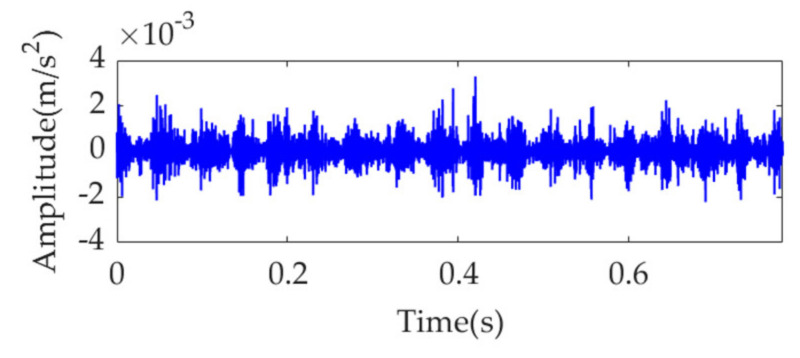
Sampled vibration dataset in the pinion fault state.

In the fault state of the pinion-tooth partial fracture, the driving motor rotation speed is set as 700 rpm. The experimental gearbox output rotation speed is 1347 rpm, as measured by the sensor. Therefore, the theoretical fault feature frequency of the pinion in the experimental gearbox is calculated as *f*_p_ = 22.45 Hz. The vibration dataset with 4000 points sampled from the experimental gearbox is shown in Figure 13.

**Figure 14:**
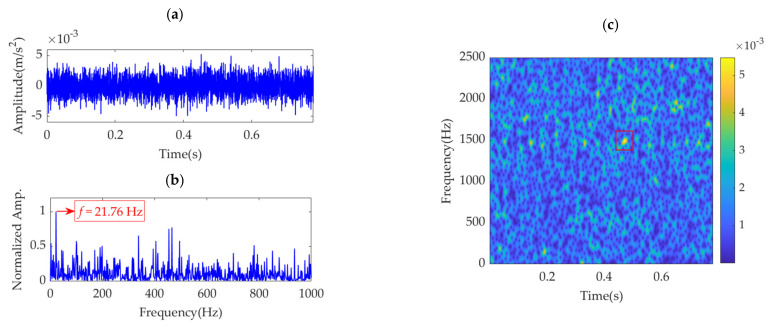
Vibration dataset with additional noise: (**a**) time–domain waveform, (**b**) SES, and (**c**) STFT spectrum.

**Figure 15:**
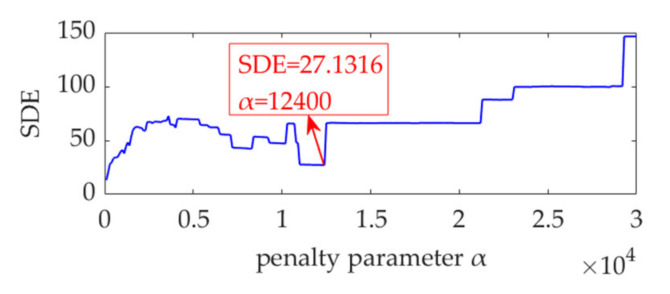
Relationship between the penalty parameter, α, and SDE index.

Because the fault impulse features exhibit significant periodicity in the time-domain waveform, analysis of this dataset may not be fruitful. To more effectively reflect the superiority of the proposed method, white Gaussian noise is added to the dataset to ensure that the signal-to-noise ratio (SNR) is −7 dB. The obtained dataset with heavy noise is shown in Figure 14a. Due to the low SNR, it is challenging to identify the impulse features in the time-domain waveform. Figure 14b shows the SES of the obtained dataset. Although a feature frequency of 21.76 Hz can be extracted using the SES, many interference components and noise are included in the SES. The STFT spectrum of the noisy dataset is shown in Figure 14c. It is challenging to identify the impulse features in the waveform. In the STFT spectrum, a few impulse features can be observed, but these features do not exhibit any regularity. Nevertheless, the strongest impulse feature appears at a position of approximately [0.4715 s, 1490 Hz], as shown in the red box of Figure 14c. Hence, the initial center frequency, *ω*_d_, of the desired mode is accordingly selected as 2π·1490 rad/s. The penalty parameter, *α*, is set to increase from 100 to 30,000, with a step size of 100. Subsequently, in each step, VME is implemented with a fixed *ω*_d_ value of 2π·1490 rad/s and an increased *α* value pertaining to the current step. Moreover, the corresponding SDE index value of the desired mode is calculated. The relationship between the penalty parameter, *α*, and SDE index is represented as a curve in Figure 15.

**Figure 16:**
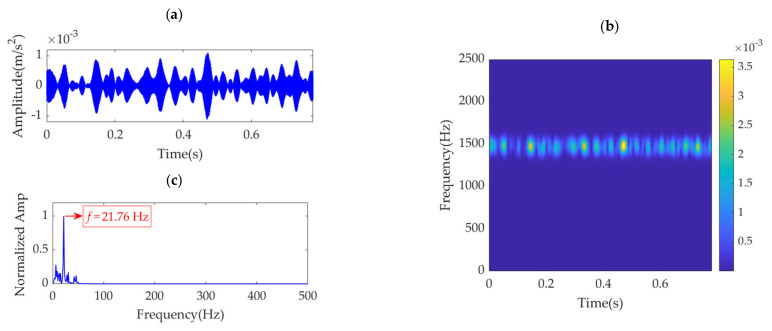
Desired mode obtained by VME with the optimal parameters: (**a**) time–domain waveform, (**b**) STFT spectrum, and (**c**) SES.

Notably, the α value of 100 corresponding to the SDE index minimum value of 13.22 is not the desired value for VME, as such a small SDE value is caused by strong random noise in the obtained desired mode. In this scenario, the optimal α value must be considered the value associated with the minimum SDE index value of 27.1316; hence, *α*_opt_ = 12,400. Accordingly, VME is implemented with an initial *ω*_d_ value of 2π·1490 Hz and an optimal α value of 12,400. The obtained desired mode and its STFT spectrum are shown in Figure 16a,b, respectively. The noise in the desired mode is substantially eliminated, and regular impulse features can be clearly observed. The SES of the desired mode is shown in Figure 16c. The feature frequency of 21.76 Hz is successfully extracted, consistent with the pinion fault feature frequency, *f*_p_.

**Figure 17:**
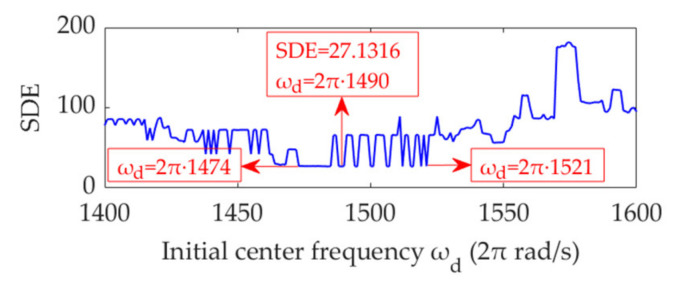
Relationship between the initial center frequency, *ω*_d_, and SDE index.

Furthermore, the penalty parameter, *α*, is set as the fixed value of *α*_opt_ = 12,400. Subsequently, the initial center frequency, *ω*_d_, is set to gradually increase from 2π·1200 rad/s to 2π·1600 rad/s in step sizes of 2π rad/s. The SDE index value of the desired mode in each step is calculated. Based on these values, the relationship curve between *ω*_d_ and the SDE index is obtained, as shown in Figure 17. The findings indicate that the initial *ω*_d_ value, 2π·1490 rad/s, selected from the STFT spectrum is the optimal value that results in a minimum SDE index value 27.1316. Notably, there exist more than one optimal initial *ω*_d_ value that minimizes the SDE index in the case of *α*_opt_ = 12,400. Moreover, the *ω*_d_ values that minimize the SDE index are not continuous. Between the *ω*_d_ values that minimize the SDE index, there are some other *ω*_d_ values that lead to large SDE index values. These values are also the optimal initial *ω*_d_ values, although their corresponding optimal *α* values must be adjusted. For instance, we set the *ω*_d_ value as 2π·1493 rad/s, leading to an SDE index value 65.7457, and the obtained optimal *α* value is an arbitrary value in the range of 12,600 to 13,000, rather than 12,400. In fact, as long as the selected *ω*_d_ value lies in the range of 2π·1474 to 2π·1521 rad/s that can be estimated by the STFT spectrum, it can be considered the optimal *ω*_d_ value. Nevertheless, its corresponding optimal *α* values may be adjusted. Therefore, there exist numerous optimal values for the parameter combination [*ω*_d_, *α*], and the proposed VME method can easily obtain a group of optimal parameters. This outcome verifies the effectiveness and robustness of the SDE index and the practicality of the proposed VME method.

**Figure 18:**
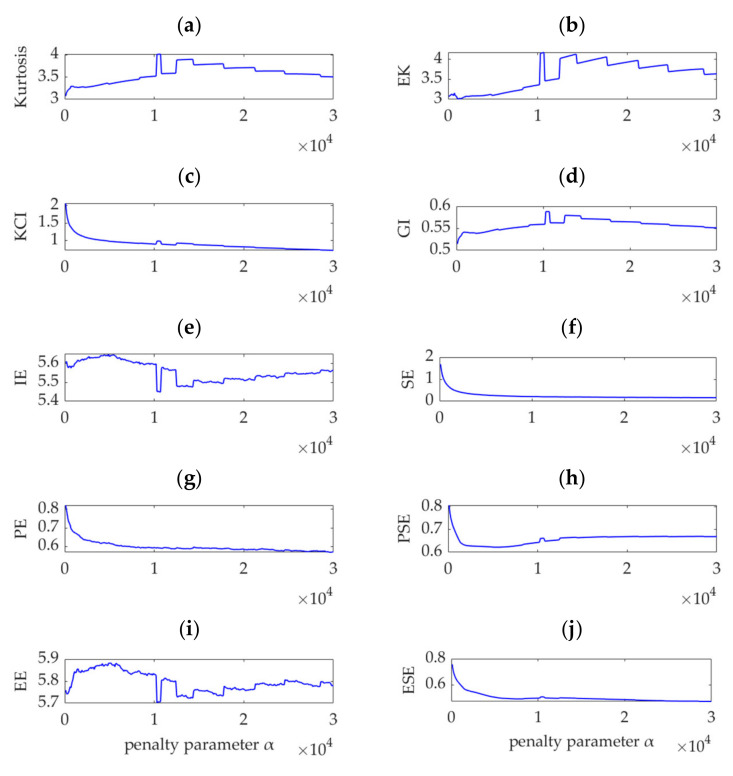
Relationship between different indices and the penalty parameter, *α*.

To verify the effectiveness of the SDE index, the 10 indices mentioned above, i.e., kurtosis, KCI, EK, GI, IE, SE, PE, PSE, EE, and ESE, for the vibration dataset in this case are calculated. As α increases, noise and interference components are first suppressed. When α is up to a certain value, i.e., the optimal value, fault features will get the best presentation. However, if α continues to increase, the fault features will be also suppressed. The relationship curves between α and the 10 indices are shown in Figure 18. Due to monotonous attenuation shown in Figure 18f,g, the SE and PE indices cannot indicate the optimal *α* values. However, as shown in Figure 18a–e,h–j, all the other indices exhibit sudden changes and achieve the maximum, minimum, or local extremum in the *α* range 10,200–10,800. In this case, the optimal *α* values indicated by these indices are not considerably different from that pertaining to the SDE index. Similarly, for the desired mode and fault feature frequency extraction, VME with the optimal *α* value indicated by the SDE index achieves the same performance as VME with the optimal *α* values indicated by the above indices. This finding demonstrates that the performance of VME is robust to the penalty parameter α. Moreover, this finding shows the effectiveness of the SDE index, although the SDE index is different from the abovementioned existing indices.

**Figure 19:**
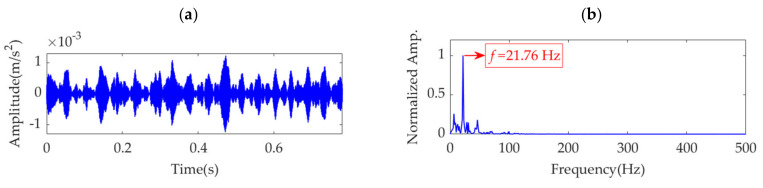
Target mode obtained by VMD: (**a**) time–domain waveform and (**b**) SES.

To verify the superiority of the improved VME method, the classical VMD method is applied to analyze the pinion fault vibration dataset. In the implementation of VMD, the penalty parameter, *α*, is set as 12,400, similar to the value in the VME method, and the mode number is set as 8, determined after several trials. By experience, the target mode is selected as the fourth mode. The time-domain waveform and SES are shown in Figure 19. Comparison of Figure 16 and Figure 19 shows that, both the improved VME method and classical VMD method can extract the pinion fault feature frequency from the vibration dataset with heavy noise. Nevertheless, the implementation of VMD may require several trials and experience, whereas the application of the improved VME method is more targeted and thus simpler and more practicable.

### 5.3. Gear Fault Vibration Dataset Analysis

**Figure 20:**
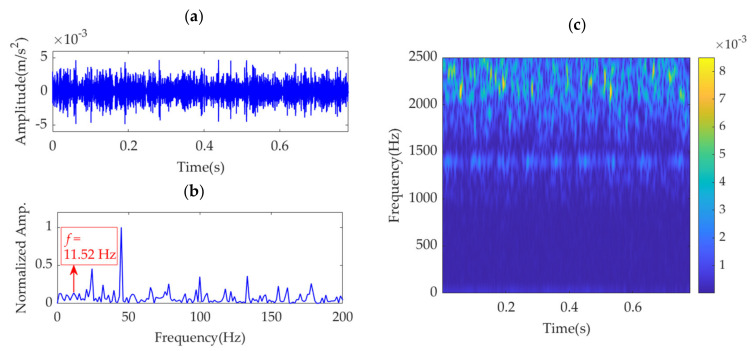
Gear fault vibration dataset: (**a**) time–domain waveform, (**b**) SES, and (**c**) STFT spectrum.

The driving motor rotation speed is set as 900 rpm, and the experimental gearbox output rotation speed is 1730 rpm, as measured by the sensor. Hence, the gear fault feature frequency, *f*_g_, in the experimental gearbox is calculated as 11.77 Hz. The vibration dataset with 4000 points sampled from the experimental gearbox is shown in Figure 20a. Because of the heavy noise, the regular impulse features cannot be recognized in the time-domain waveform. In the SES shown in Figure 20b, several fault-related spectrum lines can be observed. However, the spectrum line at the fault feature frequency cannot be identified. The STFT spectrum shown in Figure 20c indicates that intermittent but not significant fault features occur at approximately 1400 Hz along the time axis.

**Figure 21:**
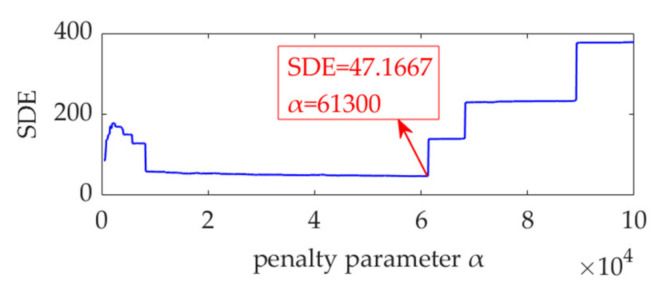
Relationship between penalty parameter α and the SDE index.

**Figure 22:**
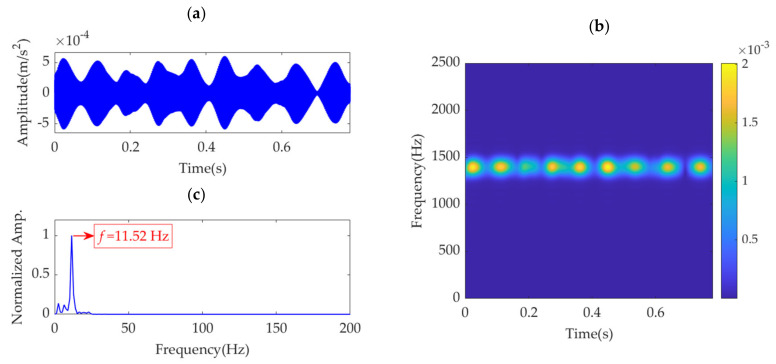
Desired mode obtained by VME with the optimal parameters: (**a**) time–domain waveform, (**b**) STFT spectrum, and (**c**) SES.

In the implementation of the improved VME method for the gear fault vibration dataset, the initial value of the center frequency, *ω*_d_, is selected as 2π·1400 rad/s. The optimal value of penalty parameter *α* is searched in the range from 500 to 100,000, with an increasing step of 100. The relationship between *α* and the SDE index is obtained and represented by the curve shown in Figure 21. The optimal α value indicated by the minimum SDE index value is *α*_opt_ = 61,300. VME with the optimal parameter group [*ω*_d_, *α*_opt_] is implemented for the vibration dataset. The obtained optimal desired mode and its STFT spectrum are shown in Figure 22a,b, respectively. The periodic fault impulse features are completely extracted with little noise. Figure 22c shows the SES of the optimal desired mode. The extracted feature frequency of 11.52 Hz is the same as the gear fault feature frequency, *f*_g_.

**Figure 23:**
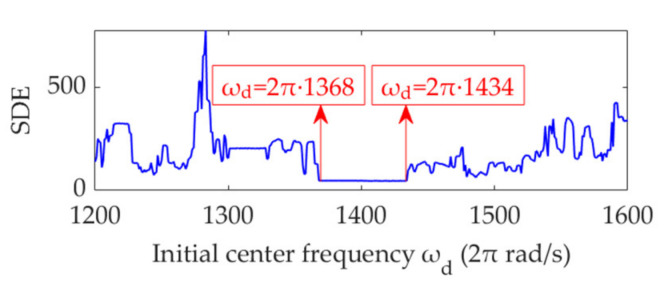
Relationship between the initial center frequency, *ω*_d_, and SDE index.

We set the penalty parameter, *α*, as a fixed value of 61300, and the initial value of the center frequency, *ω*_d_, increases from 2π·1200 rad/s to 2π·1600 rad/s, with a step size of 2π rad/s. Using the same method as mentioned previously, the relationship curves between *ω*_d_ and the SDE index are obtained, as shown in Figure 23. The value of 2π·1400 rad/s, which minimizes the SDE index, is indeed the optimal value of *ω*_d_. In addition, any value within the range of 2π·1368 rad/s to 2π·1434 rad/s can be considered the optimal initial value of *ω*_d_. This aspect suggests that the performance of VME is highly robust to the initial *ω*_d_ value. Moreover, the appropriate initial *ω*_d_ value can be selected using the STFT spectrum in gearbox fault feature extraction.

**Figure 24:**
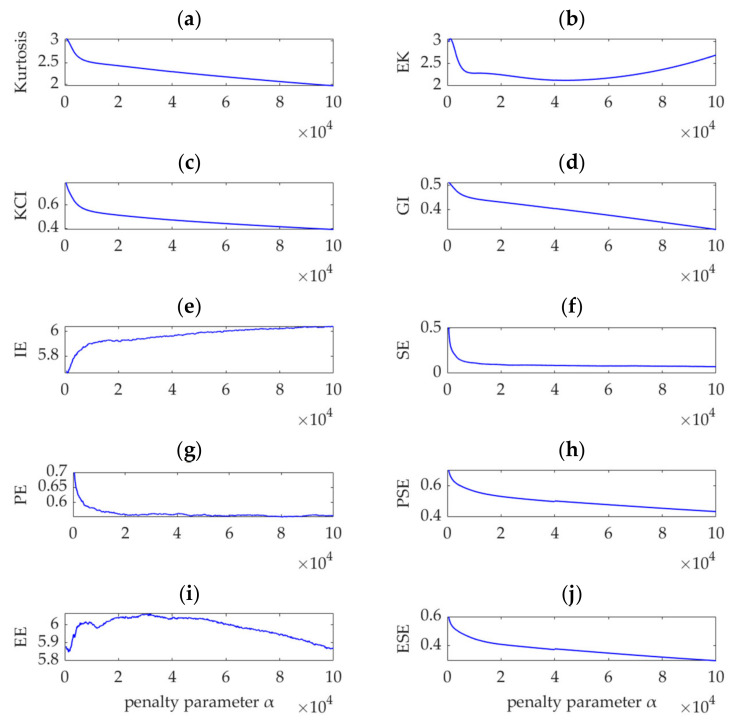
Relationship between different indices and the penalty parameter, *α*.

Like in the previous case, the 10 indices, i.e., kurtosis, KCI, EK, GI, IE, SE, PE, PSE, EE, and ESE for the gear vibration dataset are calculated, and the relationship curves between *α* and the 10 indices are obtained. Except for the EK and EE indices which are shown in Figure 24b,i, all the other indices vary monotonously with increasing *α*, as shown in Figure 24a,c–h,j, and no extrema or sudden change values are observed. Therefore, these indices cannot indicate the optimal values of α in the search range. Figure 24b shows that the EK index is minimized, and its corresponding *α* value is 44,100. Figure 24i shows that a maximum value exists for the EE index, and the corresponding *α* value is 31,400. Results of experiments show that the VME methods with different optimal *α* values indicated by the SDE, EK, and EE indices exhibit similar performance for gear fault feature extraction. However, considering the large differences between the three optimal α values, VME is highly robust to *α*, and the optimal *α* value can be selected within a large range. This aspect can be verified by the SDE index variation curve shown in Figure 21, in which the SDE index presents a nearly horizontal varying trend in the *α* range of 8,300–61,300. As the other indices do not have such an indicative function, the SDE index is considered superior.

**Figure 25:**
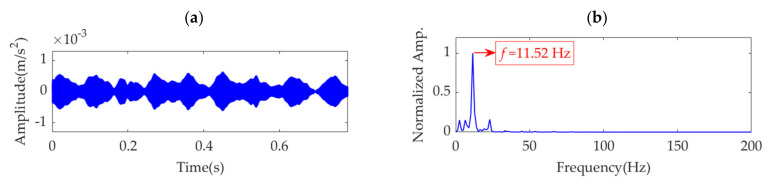
Target mode obtained using VMD: (**a**) time–domain waveform and (**b**) SES.

Furthermore, VMD is used to analyze the gear fault vibration dataset. In the implementation of VMD, its penalty parameter is set to the same value as that in the VME method, i.e., 61,300, and the number of decomposed modes is set as 8, which is an appropriate value determined by several trials. The target mode containing valuable fault feature information is the fourth mode. The corresponding time–domain waveform and SES are shown in Figure 25. Although the gear fault feature frequency can be extracted from the target mode, comparison of Figure 25 with Figure 22 shows that the desired mode obtained by VME exhibits a smaller amount of noise and fewer interference components than that in the target mode obtained by VMD.

## 6. Conclusions

VME directly decomposes a signal into the desired mode and residual signal. Compared with VMD, VME is more feasible to implement. The performance of VME is highly robust to the initial value of the desired mode center frequency and the penalty parameter. In the application of fault feature extraction and fault diagnosis for gearboxes, a reasonable initial center frequency value can be easily selected using the STFT spectrum. Moreover, the proposed SDE index can effectively indicate the optimal value of the penalty parameter and is superior to the currently widely used kurtosis-class and entropy-class indices. Accordingly, the proposed improved VME approach exhibits powerful anti-noise performance and can thus extract clear and complete fault impulse features from gearbox vibration signals with low SNRs. In addition, compared with the target mode extracted by VMD, the desired mode extracted by the improved VME approach contains fewer noise and interference components and thus achieves a superior effect for gearbox fault feature extraction. In the future, we may use the improved VME approach in feature extraction of large-scale data for gearboxes and develop intelligent fault diagnosis approaches based on deep learning.

## Figures and Tables

**Table 1:** Parameters of the simulation signal model.

*Z*	*f*_r_	*f*_m_	*N*	*X*(*n*)	*φ_n_*	*A_n_*	*α_n_*	*B_n_*	*β*_n_	*ς*	*T*
25	20 Hz	500 Hz	1	2	π	0.25	π	0.5	π	60π	0.05 s

## Data Availability

Not applicable.
